# Influence of sex and presence of cardiovascular risk factors on relations between cardiorespiratory fitness and cerebrovascular hemodynamics

**DOI:** 10.1152/japplphysiol.00371.2022

**Published:** 2022-09-08

**Authors:** Wesley K. Lefferts, Cynthia M. Weiner, Sara E. Mascone, Jacqueline A. Augustine, Kevin S. Heffernan, Elizabeth C. Lefferts

**Affiliations:** ^1^Department of Kinesiology, Iowa State University, Ames, Iowa; ^2^Department of Kinesiology, University of Maryland, College Park, Maryland; ^3^Kinesiology Department, SUNY Cortland, Cortland, New York; ^4^Department of Exercise Science, Syracuse University, Syracuse, New York

**Keywords:** aerobic exercise, cerebral pulsatility, modifiable risk factors, pulsatile damping, pulsatility

## Abstract

Cerebral hemodynamics and pulsatility are important mechanisms of cerebrovascular and brain health. Cardiorespiratory fitness may improve cerebrovascular pulsatility in healthy females, but not in males. Whether cardiovascular disease (CVD) risk factors modify sex-specific associations of fitness with cerebral hemodynamics and vascular contributors to cerebral hemodynamics is unknown. We assessed V̇o_2peak_ and cerebrovascular hemodynamics in 157 adults without (42 ± 13 yr, BMI 24.5 ± 2.7 kg/m^2^), and 66 adults with modifiable CVD risk factors (54 ± 8 yr, BMI 29.9 ± 4.0 kg/m^2^). Intracranial [middle cerebral artery (MCA) pulsatility index (PI), mean velocity, conductance, and pulsatile damping] and extracranial hemodynamics [carotid artery wave transmission/reflection, PI, pulse wave velocity (PWV)-β, and carotid-femoral PWV] were assessed via transcranial Doppler/ultrasound and tonometry. Cardiorespiratory fitness was assessed via V̇o_2peak_ during an incremental exercise test. Multiple regression was used to assess contributions of V̇o_2peak_ to cerebrovascular outcomes after adjustment for relevant covariates. V̇o_2peak_ was inversely associated with MCA PI among females (β = −0.39, *P* = 0.01) but not males (β = −0.16, *P* = 0.25) without CVD risk factors. V̇o_2peak_ was positively associated with MCA PI among females (β = 0.44, *P* = 0.01) and not associated in males with CVD risk factors (β = −0.06, *P* = 0.079). V̇o_2peak_ was beneficially associated with vascular contributors to cerebral hemodynamics but had sex-specific associations with carotid stiffness and pulse pressure in females without CVD risk factors only. These results suggest that sex-specific associations between fitness and cerebral pulsatility among females without CVD risk factors may relate to the differential effects of fitness on carotid stiffness and pulse pressure. In addition, the presence of modifiable CVD risk factors may influence the protective relations of fitness on cerebrovascular hemodynamics.

**NEW & NOTEWORTHY** We identify beneficial associations between cardiorespiratory fitness and lower carotid stiffness and pulse pressure as potential mechanisms underlying sex-specific associations of fitness and cerebral pulsatility in females without modifiable risk factors. Greater fitness is beneficially associated with conductance, pulsatile damping, and forward wave energy among adults without risk factors; however, associations are attenuated among adults with modifiable risk factors. These data suggest sex and risk factors may alter cerebrovascular sensitivity to cardiorespiratory fitness.

## Graphical abstract

**Figure:**
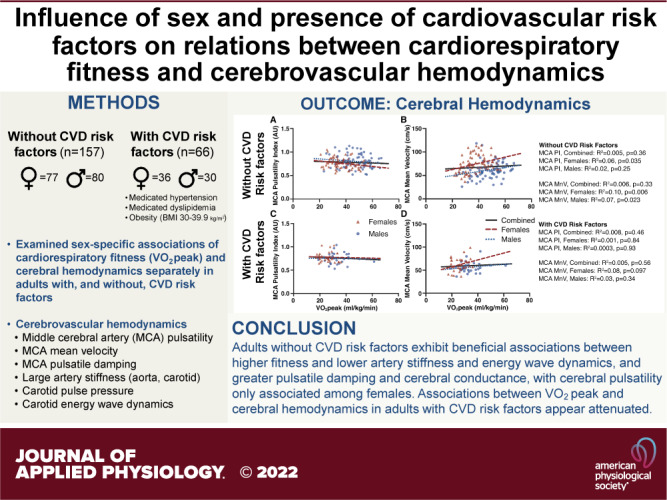

## INTRODUCTION

Regular aerobic exercise increases cardiorespiratory fitness, which is linked to improved brain health with aging. As such, regular aerobic exercise has emerged as a critical lifestyle behavior to improve vascular mechanisms of later-life cerebrovascular and cognitive disease (1). Maintaining cardiorespiratory fitness for a given age protects against age-related brain atrophy and damage (2–4), and cognitive dysfunction (5, 6). The exact mechanisms through which regular aerobic exercise and cardiorespiratory fitness protect the brain remain elusive but may stem in part from changes in cerebral blood flow.

Regular aerobic exercise may optimize cerebral blood flow by increasing blood flow and conductance and reducing cerebral pulsatility. Indeed, higher cardiorespiratory fitness is associated with improved (i.e., greater) cerebral blood velocity/flow (7, 8), cerebrovascular conductance (8, 9), and reduced cerebral pulsatility (10, 11), all of which are typically impaired with aging (9, 12) and in the presence of cognitive disease (13). Recently, Zeller et al. (11) noted sex-specific associations between greater cardiorespiratory fitness (i.e., V̇o_2peak_) and lower middle cerebral artery pulsatility among females after adjusting for age, but not in the sample as a whole or in males alone. Moreover, they noted no beneficial association between cardiorespiratory fitness and cerebral blood velocity. The work by Zeller et al. (11) was a critical first step to draw attention to the influence of sex on cardiorespiratory fitness and cerebral blood velocity and pulsatility across the lifespan; however, the analysis included a narrow appraisal of cerebral hemodynamics and was limited to a sample of markedly healthy adults free from modifiable cardiovascular disease (CVD) risk factors and with a limited distribution of middle-aged adults. It is now recognized that stiffness and function of the large arteries outside of the brain (e.g., aorta and common carotid) influence intracranial cerebral hemodynamics by altering how blood pressure/flow is transmitted/reflected en route to the cerebrovasculature (12) and thus, are highly related to brain health and cognitive function (14–17). As such, vascular contributors to cerebral hemodynamics may play an additional mechanistic role in mediating the benefits of cardiorespiratory fitness on the brain and sex-dependent associations of fitness and cerebral hemodynamics. Although healthy populations are ideal for identifying initial relationships between physiological signals of interest, they are not the populations at greatest risk of later-life cognitive/cerebrovascular disease. Indeed, CVD risk factors (e.g., hypertension, dyslipidemia, and obesity) can impair cerebrovascular hemodynamics (1, 18, 19) and common pharmaceutical treatments for these risk factors may influence exercise adaptations (20). As such, presence of CVD risk factors may alter associations between cardiorespiratory fitness and cerebrovascular hemodynamics.

The aims of this study are threefold: *1*) to reproduce recent findings of Zeller et al. (11) regarding sex-specific relations of cardiorespiratory fitness (V̇o_2peak_) and middle cerebral artery (MCA) mean velocity and pulsatility in adults without modifiable CVD risk factors across the lifespan, and expand upon the findings of Zeller et al. (11) by *2*) performing the same analyses in a sample of adults with modifiable CVD risk factors (obesity, medicated hypertension/dyslipidemia), and *3*) examine sex-specific relations of V̇o_2peak_ and novel vascular contributors to cerebral hemodynamics (pulsatile damping, large artery stiffness, wave transmission/reflection, and pulse pressure) identified in our previous work (12) in both males and females with, and without, modifiable CVD risk factors. We hypothesized that relations of cardiorespiratory fitness with cerebrovascular hemodynamics and vascular contributors would be attenuated among males and females with CVD risk factor, and that greater cardiorespiratory fitness would be associated with greater pulsatile damping, lower large artery stiffness, pulse pressure, wave transmission, and greater wave reflection in females, but not in males, without CVD risk factors.

## METHODS

### Participants

This study is a new analysis of previously published (12, 21–25) and unpublished data (*n* = 47) collected from 223 adults between the ages of 18 and 72 yr old (50.7% female) between 2017 and 2022. Data were collected across multiple institutions, including Syracuse University (*n* = 117), the University of Illinois at Chicago (*n* = 59), and Iowa State University (*n* = 47). All participants were generally healthy, as previously described (12, 21–25), and individuals were excluded if they were current smokers, had class III obesity [body mass index (BMI) >40 kg/m^2^], diabetes mellitus, or diagnosed with cardiovascular disease/previous events. For the purpose of this analysis, participants were classified based on apparent absence or presence of certain modifiable CVD risk factors (operationally defined later). Adults without CVD risk factors were operationally defined based on criteria defined by Zeller et al. (11) where participants were “considered healthy based on cardiovascular disease risk classification from the American College of Sports Medicine (ACSM) criteria.” Accordingly, we categorized participants as free from modifiable CVD risk factors if they were nonobese (BMI < 30 kg/m^2^), had no known disease history, and were not taking antihypertensive or lipid-lowering medication. Adults with modifiable CVD risk factors were operationally defined by the presence of one or more of the following: obesity (BMI ≥ 30.0 kg/m^2^), diagnosed with dyslipidemia or hypertension [assessed via use of lipid-lowering medication (i.e., statin), or antihypertensive medication]. We did not directly assess all CVD risk factors (e.g., family history of CVD and plasma lipid levels), and by design certain CVD risk factors were excluded (e.g., diabetes and smoking). Participants with high resting blood pressure were not categorized as hypertensive unless they reported the use of antihypertensive medication since single-point office blood pressure measures are not sufficient to accurately diagnose hypertension (26). All participants provided written informed consent, and all study procedures were approved by the institutional review board at their respective institutions (Syracuse University, University of Illinois at Chicago, and Iowa State University) and conformed to the standards outlined in the Declaration of Helsinski.

### Study Design

Information retrieved from the respective studies include demographic (age, sex, and menopause status), self-reported current exercise habits, body composition (height, weight, and BMI), cardiorespiratory fitness (V̇o_2peak_), and resting cerebrovascular hemodynamics. Pre-, peri-, and postmenopausal status was documented using the STRAW+ 10 guidelines (27). Cerebrovascular hemodynamics were assessed following ≥4 h fast and abstinence from exercise/caffeine the day of testing. All cerebrovascular measures were taken in the supine position in a dimly lit room after at least 10 min of quiet rest/instrumentation. Premenopausal (*n* = 70) and perimenopausal females (*n* = 12) were tested during the early follicular phase of their menstrual cycle or during the placebo pill phase if taking oral contraceptives. Measurement periods for males (*n* = 110) and postmenopausal females (*n* = 31) were not standardized. No females were using hormone replacement therapy.

### Measurements

#### Cardiorespiratory fitness.

Participants completed a maximal exercise test to measure peak oxygen consumption (V̇o_2peak_). V̇o_2peak_ was assessed via an incremental exercise test on a treadmill (*n* = 177) or electronically braked cycle ergometer (*n* = 46). Ventilation and gas exchange was assessed using a TrueOne [Parvo Medics, Sandy, UT (*n* = 176)] or Moxus [AEI Technologies, Pittsburgh, PA (*n* = 47)] metabolic cart. V̇o_2peak_ was determined using a 15-s epoch and considered valid if two of the three following criteria were achieved: heart rate (HR) peak within 10 beats/min of age-predicted maximal heart rate (220 − age); respiratory exchange ratio (RER) ≥ 1.10; RPE ≥ 17 on Borg scale.

### Cerebrovascular Hemodynamics

#### Middle cerebral artery hemodynamics.

MCA mean blood velocity and pulsatility index (PI) were measured via Transcranial Doppler (DWL Doppler Box-X, Compumedics, Germany; *n* = 117; TOCM Nuerovision, MultiGON Industries, Elmsford, NY; *n* = 106) as described previously (12, 25). Briefly, the MCA was insonated and confirmed via standard criteria (28) using a 2-MHz transcranial probe secured to the left temporal window. MCA mean velocity (MnV) and PI were calculated over a 24- to 120-s epoch. MCA PI was calculated using peak systolic (*V*_s_), diastolic (*V*_d_), and MnV as PI = (*V*_s_ *– V*_d_)/MnV. In line with our prior work (12, 25), cerebral pulsatile damping was calculated as the ratio of proximal (carotid) to distal (MCA) pulsatility. MCA conductance was calculated as MCA MnV/brachial mean arterial pressure. End-tidal CO_2_ (ETCO_2_) was measured during cerebrovascular assessments (23, 25) in a subset of participants (*n* = 132) via facemask (*n* = 39, MP150, BioPac Systems, Goleta, CA), nasal cannula (*n* = 46, Nellcor OxiMax, Covidien, Mansfield, MA), or mouthpiece (*n* = 47, AGM100, MediPines, Yorba Linda, CA).

#### Aortic stiffness.

Aortic stiffness was measured using carotid-femoral (cf) pulse wave velocity (PWV), the “gold standard” for central arterial stiffness measurements (29) as described previously (12, 25). Briefly, applanation tonometry (AtCor Medical, Sydney, Australia, *n* = 117; NIHem 2.0, Cardiovascular Engineering Inc., Norwood, MA, *n* = 106) was used to measure femoral and carotid blood pressure waveforms over a 10- to 20-s epoch with simultaneous R wave gating. CfPWV was calculated as the transit distance between the carotid and femoral sites divided by the time delay between peak R-wave and the foot of the corresponding pressure waveform.

#### Carotid hemodynamics.

Common carotid artery hemodynamics were assessed just upstream of the carotid bulb via ultrasound (Aloka ProSound α7, *n* = 156; Arietta 70, *n* = 46; Hitachi Healthcare Americas, Twinsburg, OH) and onboard eTracking software using a 7.5- to 10.0-Hz linear-array probe, as described previously in detail (12, 25). Carotid stiffness was calculated using a local single-point PWV (PWV-β) calibrated to carotid systolic/diastolic blood pressure (described below in *Blood pressure* section) and PI was calculated in the same manner as MCA PI. Carotid wave intensity analysis was conducted using blood pressure (*P*) and velocity (*U*) in relation to time, with WI = (d*P*/d*t* × d*U*/d*t*) (12). Wave intensity analysis allows derivation of *W*1, a forward-traveling compression wave during systole that drives blood flow and contributes to pulsatility, and negative area, a backward-traveling compression wave that adds to pressure but detracts from flow (12). The ratio of negative area to *W*1 is used to determine the reflection index (RIx), which we have linked with cerebral pulsatile damping (12).

#### Blood pressure.

Brachial systolic and diastolic blood pressure were measured via oscillometry or finger photoplethysmography calibrated to brachial blood pressure. Brachial mean arterial pressure was calculated as 1/3 systolic + 2/3 diastolic pressure. A 10- to 20-s epoch of carotid pressure waves were ensemble averaged and calibrated to brachial mean and diastolic pressure as described previously (12, 25).

### Statistical Analysis

Statistical analyses were performed in SPSS v28 (IBM, Chicago, IL) and graphing was performed via GraphPad Prism version 9 (GraphPad, San Diego, CA). For all analyses, statistical significance was set a priori as α < 0.05. Participant descriptive characteristics, self-reported current exercise habits (see Supplemental Content), and resting cerebrovascular hemodynamics were compared between males and females using two-tailed, independent sample *t* tests for adults with and without CVD risk factors, separately.

Univariate regressions were performed to examine associations between V̇o_2peak_ and MCA hemodynamics. Multiple regression was performed to explore relations of V̇o_2peak_ with MCA hemodynamics and novel vascular contributors to cerebral hemodynamics separately in adults with and without CVD risk factors, as well as by sex within each group. Novel vascular contributors were selected based on our prior work that identified cerebral pulsatile damping, large artery (carotid, aortic) stiffness, forward wave energy transmission (*W*1)/reflection (RIx), and carotid pulse pressure as contributors to MCA PI (12), and substantial body of literature linking mean arterial pressure to MCA mean velocity (30). Multiple regression analyses examining relations between V̇o_2peak_ and cerebral hemodynamics among adults without CVD risk factors are adjusted for age (*model 1*); and age, BMI, female sex, and modality (for V̇o_2peak_ assessment) (*model 2*). Covariates included in the combined and sex-specific analyses among the CVD risk factor group were similar but excluded modality since this variable inadvertently adjusted for hypertension medication [nearly all hypertensive individuals in this sample performed their V̇o_2peak_ test on a cycle ergometer (24)]. For this reason, and since there was no difference in the distribution of males and females who performed cycle versus treadmill tests in this group, we decided to exclude covariate adjustment for modality in the models for adults with CVD risk factors.

In addition, following Zeller et al. (11), we built models including age, sex, V̇o_2peak_, and three- and two-way interactions between the covariates with MCA mean velocity and PI as dependent variables. Models were reduced until significance was achieved, with the three-way interactions being removed first, followed by the two-way interactions, keeping age and sex at a minimum. No interaction terms were significant at any step for either MCA mean velocity or PI (data not shown).

## RESULTS

### Descriptive Characteristics of Samples With and Without CVD Risk Factors

The cohort without CVD risk factors contained 157 participants (77 females, 80 males) with 57 pre-, 9 peri-, and 11 postmenopausal females. Eleven of the premenopausal females were taking oral contraceptives and 46 were naturally menstruating. Males without CVD risk factors exhibited significantly higher BMI, V̇o_2peak_, brachial and carotid systolic pressure, carotid pulse pressure, MCA and carotid pulsatility, MCA pulsatile damping, and cfPWV, and lower MCA mean velocity and conductance than females without CVD risk factors (Table 1). The cohort with CVD risk factors contained 66 participants (36 females, 30 males) with 13 pre-, 3 peri-, and 20 postmenopausal females. Two of the premenopausal females were on birth control (1 oral contraceptive, 1 intrauterine device) and 11 were naturally menstruating. Males with CVD risk factors had higher V̇o_2peak_, BMI, and exhibited greater MCA pulsatile damping, carotid pulsatility, and lower MCA mean velocity than females with CVD risk factors. Self-reported exercise habits in each cohort are reported in Supplemental Table S1 and were not statistically different between males and females without CVD risk factors, or between males and females with CVD risk factors.

**Table 1.** Descriptive characteristics and cerebrovascular hemodynamics for individuals with and without CVD risk factors

	Without CVD Risk Factors			Female	Male	With CVD Risk Factors			Female	Male
	Combined	Female	Male	*n*	*n*	Combined	Female	Male	*n*	*n*
Age, yr	42 ± 13	41 ± 12	43 ± 13	77	80	54 ± 8	53 ± 9	55 ± 8	36	30
Height, m	1.71 ± 0.10	1.64 ± 0.07	1.77 ± 0.07*	77	80	1.71 ± 0.09	1.65 ± 0.07	1.79 ± 0.06*	36	30
Weight, kg	71.9 ± 11.9	65.0 ± 8.7	78.5 ± 10.7*	77	80	87.4 ± 15.0	82.2 ± 12.6	93.6 ± 15.6*	36	30
BMI, kg/m^2^	24.5 ± 2.7	24.0 ± 2.7	24.9 ± 2.5*	77	80	29.9 ± 4.0	30.3 ± 3.8	29.3 ± 4.1	36	30
V̇o_2peak_, mL/kg/min	42.1 ± 10.7	37.1 ± 8.8	46.9 ± 10.1*	77	80	29.6 ± 7.9	26.0 ± 5.2	33.9 ± 8.5*	36	30
V̇o_2peak_, L/min	3.0 ± 0.9	2.4 ± 0.5	3.6 ± 0.8*	77	80	2.6 ± 0.8	2.1 ± 0.5	3.1 ± 0.8*	36	30
Fitness level, %ile	74 ± 25	73 ± 27	74 ± 22	77	80	58 ± 24	57 ± 23	59 ± 25	36	30
SP, mmHg	120 ± 13	116 ± 15	124 ± 10*	77	80	123 ± 23	118 ± 28	129 ± 12*	36	30
DP, mmHg	74 ± 11	74 ± 13	74 ± 8	77	80	84 ± 16	87 ± 20	81 ± 9	36	30
MAP, mmHg	91 ± 8	89 ± 9	92 ± 8	77	80	98 ± 10	98 ± 12	98 ± 9	36	30
MCA mean velocity, cm/s	57 ± 10	60 ± 9	55 ± 10*	75	77	61 ± 10	64 ± 10	58 ± 9*	36	30
MCA PI, au	0.78 ± 0.13	0.76 ± 0.11	0.80 ± 0.14*	75	77	0.77 ± 0.11	0.78 ± 0.10	0.75 ± 0.11	36	30
MCA conductance, cm/s/mmHg	0.75 ± 0.22	0.84 ± 0.22	0.67 ± 0.19*	75	77	0.61 ± 0.15	0.64 ± 0.16	0.58 ± 0.12	36	30
MCA pulsatile damping, au	1.91 ± 0.47	1.76 ± 0.41	2.05 ± 0.49*	74	76	1.75 ± 0.35	1.58 ± 0.30	1.96 ± 0.30*	36	30
End-tidal CO_2_, mmHg	37 ± 5	36 ± 6	37 ± 5	37	39	37 ± 5	37 ± 5	36 ± 4	32	23
cf PWV, m/s	6.8 ± 1.5	6.5 ± 1.2	7.1 ± 1.6*	77	80	8.0 ± 1.4	7.9 ± 1.3	8.0 ± 1.6	36	30
Carotid PWV-β, m/s	5.4 ± 1.0	5.2 ± 1.1	5.5 ± 1.0	77	79	6.3 ± 1.2	6.3 ± 1.3	6.3 ± 1.2	36	30
Carotid *W*1, mmHg/m/s^3^	8.5 ± 5.0	8.0 ± 5.2	9.0 ± 4.8	77	78	7.2 ± 3.7	7.1 ± 4.0	7.4 ± 3.3	36	30
Carotid RIx, au	4.7 ± 2.2	5.0 ± 2.4	4.5 ± 1.9	77	78	4.3 ± 2.0	4.2 ± 2.1	4.4 ± 1.9	35	30
Carotid PI, au	1.46 ± 0.37	1.39 ± 0.29	1.61 ± 0.38*	76	79	1.33 ± 0.25	1.22 ± 0.23	1.45 ± 0.22*	35	30
Carotid SP, mmHg	115 ± 13	113 ± 15	117 ± 11*	77	80	121 ± 15	121 ± 16	121 ± 12	36	30
Carotid PP, mmHg	41 ± 11	40 ± 13	42 ± 9	77	80	40 ± 11	40 ± 11	39 ± 11	36	30

Means ± SD. BMI, body mass index; cf, carotid-femoral; CVD, cardiovascular disease; DP, diastolic pressure; MAP, mean arterial pressure; MCA, middle cerebral artery; PI, pulsatility index; PP, pulse pressure; PWV, pulse wave velocity; RIx, reflection index; SP, systolic pressure; V̇o_2peak_, peak oxygen consumption; *W*1, forward wave energy.

* *P* < 0.05 vs. female within group. Statistical differences not tested between those with and without CVD risk factors.

### Reproducibility of Sex-Specific Associations of Cardiorespiratory Fitness and MCA Pulsatility and Mean Velocity in Adults Without CVD Risk Factors

**Figure 1.**
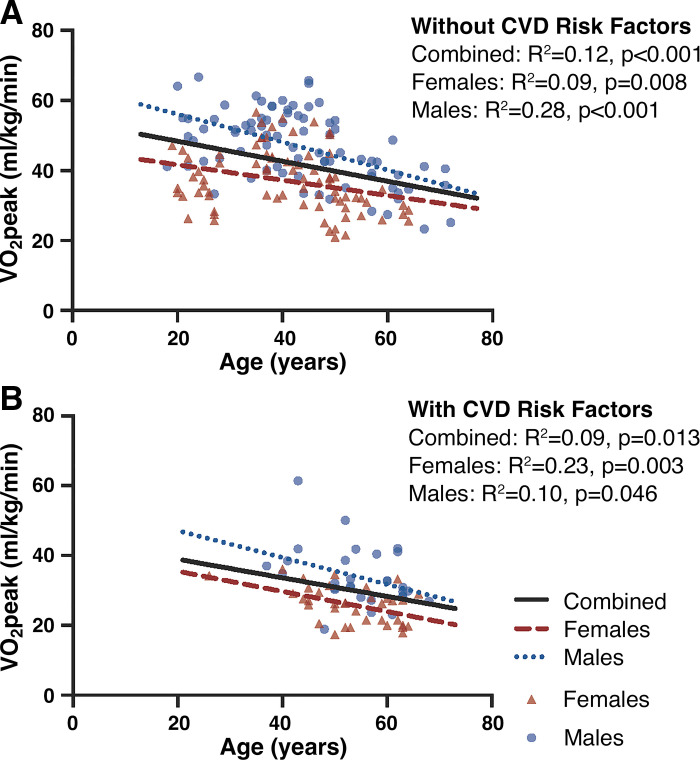
Association between cardiorespiratory fitness (V̇o_2peak_) and age in *n* = 157 adults without cardiovascular disease (CVD) risk factors (*A*), and *n* = 66 adults with CVD risk factors (*B*). Females displayed in red triangles, males in blue circles.

**Figure 2.**
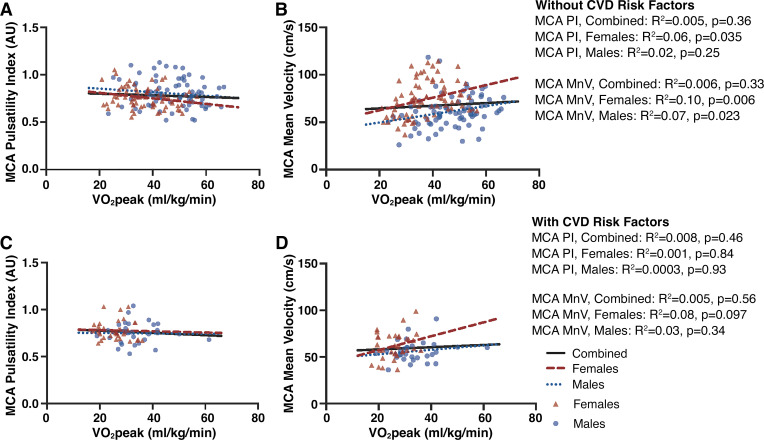
Association between cardiorespiratory fitness (V̇o_2peak_) and middle cerebral artery (MCA) pulsatility index (*A* and *C*) and MCA mean velocity (*B* and *D*). Adults without cardiovascular disease (CVD) risk factors (*n* = 152) are displayed in *A* and *B*, and adults with CVD risk factors (*n* = 66) in *C* and *D*. Females displayed in red triangles, males in blue circles. CVD, cardiovascular disease; MnV, MCA mean velocity; PI, pulsatility index.

V̇o_2peak_ was negatively associated with age within the entire cohort without CVD risk factors (Fig. 1). Unadjusted univariate analyses revealed that although V̇o_2peak_ was not associated with MCA PI or mean velocity in the combined sample (Fig. 2), V̇o_2peak_ was inversely associated with MCA PI in females only (*P* = 0.035, Fig. 2*A*), and positively associated with MCA mean velocity in both males (*P* = 0.023) and females (*P* = 0.006; Fig. 2*B*). In the combined male and female sample, V̇o_2peak_ and sex were significant predictors of MCA PI after adjusting for covariates (Table 2). In the fully adjusted sex-specific analyses, V̇o_2peak_ significantly contributed to MCA PI in females, but not in males. V̇o_2peak_ was not related to MCA mean velocity in the combined sample or males only. Among females, however, V̇o_2peak_ was positively associated with MCA mean velocity after adjusting for age (*P* = 0.01) but was no longer significant after further adjustment for BMI and modality (*P* = 0.13).

**Table 2.** Multiple regression assessing association between cardiorespiratory fitness (V̇o_2peak_) and cerebral hemodynamics in males and females without CVD risk factors

Model	Without CVD Risk Factors					
	Combined		Female		Male	
	*B* (95% CI)	β	*B* (95% CI)	β	*B* (95% CI)	β
MCA PI						
1						
V̇o_2peak_	−0.001 (−0.003, 0.001)	−0.07	−**0.003 **(−**0.006, 0.000)**	−**0.28**	−0.002 (−0.006, 0.002)	−0.14
Age	0.000 (−0.002, 0.002)	0.01	−0.001 (−0.003, 0.001)	−0.12	0.000 (−0.003, 0.003)	−0.01
2						
V̇o_2peak_	−**0.003 **(−**0.006, 0.000)**	−**0.24**	**−0.005 **(−**0.008,** −**0.001)**	−**0.39**	−0.002 (−0.006, 0.002)	−0.16
Age	−0.001 (−0.003, 0.001)	−0.08	0.000 (−0.003, 0.002)	−0.05	−0.001 (−0.004, 0.002)	−0.09
BMI	−0.003 (−0.012, 0.006)	−0.06	−0.003 (−0.003, 0.002)	−0.09	−0.006 (−0.020, 0.008)	−0.11
Modality	−0.038 (−0.119, 0.043)	−0.08	**0.119 (0.008, 0.231)**	**0.26**	−**0.125 **(−**0.242,** −**0.008)**	−**0.27**
(Female sex)	−**0.074 **(−**0.125,** −**0.023)**	−**0.29**				
MCA mean velocity						
1						
V̇o_2peak_	−0.05 (−0.332, 0.232)	−0.03	**0.495 (0.035, 0.955)**	**0.24**	0.26 (−0.175, 0.695)	0.16
Age	−**0.51 **(−**0.751,** −**0.269)**	−**0.34**	−**0.475 **(−**0.819,** −**0.130)**	−**0.30**	−0.253 (−0.587, 0.080)	−0.2
2						
V̇o_2peak_	0.313 (−0.035, 0.660)	0.18	0.484 (−0.137, 1.105)	0.23	0.222 (−0.222, 0.667)	0.13
Age	−**0.366 **(−**0.611,** −**0.121)**	−**0.24**	−**0.445 **(−**0.811,** −**0.08)**	−**0.28**	−**0.352 **(−**0.696,** −**0.007)**	−**0.27**
BMI	−0.397 (−1.590, 0.797)	−0.06	0.251 (−1.762, 2.263)	0.04	−0.716 (−2.299, 0.867)	−0.11
Modality	−5.159 (−15.897, 5.579)	−0.08	9.768 (−8.876, 28.413)	0.12	−**13.677 **(−**26.909,** −**0.444)**	−**0.25**
(Female sex)	**14.718 (7.945, 21.492)**	**0.39**				

BMI, body mass index; CVD, cardiovascular disease; MCA, middle cerebral artery; PI, pulsatility index; V̇o_2peak_, peak oxygen consumption. **Bold** denotes significant contributor at *P* < 0.05. Female (i.e., sex) was only included as a covariate in the combined analysis. Males, *n* = 77; females, *n* = 75.

### Association of Cardiorespiratory Fitness and MCA Pulsatility and Mean Velocity in Adults with CVD Risk Factors

Unadjusted associations between V̇o_2peak_ and age, MCA PI, and MCA mean velocity among males and females with modifiable CVD risk factors are displayed in Fig. 1*B* and Fig. 2, *C*and *D*, respectively. Univariate analyses revealed V̇o_2peak_ was inversely associated with age (*P* = 0.046–0.003) but was not associated with MCA PI or mean velocity among males and females (combined or separated by sex). V̇o_2peak_ was not associated with MCA mean velocity in the combined sample or separately among males or females in the fully adjusted model, however, V̇o_2peak_ was positively associated with MCA pulsatility in females only (*P* = 0.01; Table 3).

**Table 3.** Multiple regression assessing association between cardiorespiratory fitness (VO_2_peak) and cerebral hemodynamics in males and females with CVD risk factors

Model	With CVD Risk Factors					
	Combined		Female		Male	
	*B* (95% CI)	β	*B* (95% CI)	β	*B* (95%CI)	*β*
MCA PI						
1						
V̇o_2peak_	0.000 (−0.003, 0.003)	0.00	0.005 (−0.002, 0.011)	0.26	0.001 (−0.005, 0.006)	0.05
Age	**0.004 (0.001, 0.007)**	**0.32**	**0.007 (0.003, 0.011)**	**0.62**	0.003 (−0.003, 0.008)	0.18
2						
V̇o_2peak_	0.002 (−0.002, 0.007)	0.17	**0.008 (0.002, 0.015)**	**0.44**	−0.001 (−0.007, 0.005)	−0.06
Age	**0.005 (0.001, 0.009)**	**0.41**	**0.010 (0.006, 0.014)**	**0.90**	0.000 (−0.006, 0.007)	0.02
BMI	0.001 (−0.006, 0.009)	0.05	**0.012 (0.004, 0.020)**	**0.47**	−0.009 (−0.021, 0.004)	−0.31
(Female sex)	0.054 (−0.007, 0.115)	0.25				
MCA mean velocity						
1						
V̇o_2peak_	0.002 (−0.417, 0.421)	0.00	0.300 (−0.671, 1.271)	0.11	0.309 (−0.240, 0.857)	0.23
Age	−0.364 (−0.76, 0.032)	−0.20	−0.557 (−1.141, 0.028)	−0.35	0.204 (−0.373, 0.780)	0.15
2						
V̇o_2peak_	0.106 (−0.408, 0.620)	0.07	0.048 (−0.992, 1.088)	0.02	0.178 (−0.395, 0.752)	0.13
Age	−**0.465 **(−**0.922,** −**0.008)**	−**0.30**	−**0.795 **(−**1.483,** −**0.106)**	−**0.50**	0.000 (−0.643, 0.643)	0.00
BMI	−0.853 (−1.747, 0.042)	−0.30	−0.875 (−2.246, 0.495)	−0.24	−0.804 (−1.991, 0.383)	−0.29
(Female sex)	6.593 (−0.731, 13.917)	0.26				

BMI, body mass index; CVD, cardiovascular disease; MCA, middle cerebral artery; PI, pulsatility index; V̇o_2peak_, peak oxygen consumption. **Bold** denotes significant contributor at *P* < 0.05. Parentheses around predictor denote they are not included in sex-specific models. Modality was not included as covariate in analyses for adults without CVD risk factor since it accounted for hypertension diagnosis. Males, *n* = 30; females, *n* = 36.

### Association between Cardiorespiratory Fitness and Novel Vascular Contributors to Cerebral Hemodynamics in Adults With and Without CVD Risk Factors

#### Adults without CVD risk factors.

V̇o_2peak_ was positively associated with MCA conductance (*P* = 0.01) and pulsatile damping (*P* < 0.001) in the combined sample of males and females (Table 4). Although V̇o_2peak_ was not associated with MCA conductance among males or females separately, higher V̇o_2peak_ was associated with greater pulsatile damping in both sex-specific analyses (females *P* = 0.04, males *P* < 0.001). V̇o_2peak_ was inversely associated with cf PWV (*P* = 0.003) and carotid PWV-β (*P* = 0.003) in the combined sample of males and females. In sex-specific associations, V̇o_2peak_ was inversely associated with cfPWV only among males (*P* = 0.01), and carotid PWV-β only among females (*P* = 0.01). V̇o_2peak_ was inversely associated with carotid forward wave energy (*W*1) in the combined sample (*P* = 0.001), and separately among males (*P* = 0.04) and females (*P* = 0.01). V̇o_2peak_ was not associated with carotid pulse pressure in the combined sample, however, an inverse association was observed among females (*P* = 0.02), but not males. V̇o_2peak_ was inversely associated with mean arterial pressure in the combined sample only (*P* = 0.02) and was unrelated to carotid wave reflection (RIx) in all analyses.

**Table 4.** Association between cardiorespiratory fitness (V̇o_2peak_) and novel vascular contributors to cerebral hemodynamics after adjustment for covariates within males and females with and without CVD risk factors

	Without CVD Risk Factors			With CVD Risk Factors		
	*n*	*B* (95% CI)	β	*n*	*B* (95% CI)	β
MCA conductance						
Combined	152	**0.005 (0.001, 0.009)**	**0.24**	66	0.002 (−0.004, 0.007)	0.09
Females	75	0.006 (−0.001, 0.014)	0.26	36	0.000 (−0.013, 0.012)	−0.01
Males	77	0.004 (−0.001, 0.009)	0.20	30	0.003 (−0.003, 0.009)	0.21
MCA pulsatile damping						
Combined	150	**0.018 (0.010, 0.027)**	**0.42**	66	−0.009 (−0.021, 0.002)	−0.21
Females	74	**0.013 (0.000, 0.026)**	**0.29**	36	−0.013 (−0.035, 0.010)	−0.22
Males	76	**0.024 (0.012, 0.036)**	**0.50**	30	−0.007 (−0.021, 0.006)	−0.21
Aortic stiffness (cf-PWV)						
Combined	157	−**0.035 **(−**0.058,** −**0.012)**	−**0.26**	66	−**0.076 **(−**0.129,** −**0.024)**	−**0.43**
Females	77	−0.022 (−0.056, 0.012)	−0.16	36	−0.079 (−0.170, 0.012)	−0.33
Males	80	−**0.042 **(−**0.075,** −**0.008)**	−**0.26**	30	−**0.076 **(−**0.149,** −**0.003)**	−**0.41**
Carotid stiffness (PWV-β)						
Combined	156	−**0.024 **(−**0.040,** −**0.008)**	−**0.24**	66	−0.008 (−0.058, 0.042)	−0.05
Females	77	−**0.036 **(−**0.064,** −**0.008)**	−**0.29**	36	−0.007 (−0.107, 0.093)	−0.03
Males	79	−0.015 (−0.035, 0.004)	−0.15	30	−0.009 (−0.071, 0.052)	−0.07
Forward wave energy (*W*1)						
Combined	155	−**0.160 **(−**0.251,** −**0.069)**	−**0.34**	65	0.066 (−0.095, 0.227)	0.14
Females	77	−**0.192 **(−**0.345,** −**0.038)**	−**0.33**	35	0.281 (−0.073, 0.635)	0.35
Males	78	−**0.128 **(−**0.250,** −**0.007)**	−**0.27**	30	−0.012 (−0.179, 0.156)	−0.03
Carotid pulse pressure						
Combined	157	−0.223 (−0.448, 0.001)	−0.22	66	0.418 (−0.049, 0.884)	0.30
Females	77	−**0.481 **(−**0.894,** −**0.068)**	−**0.33**	36	0.512 (−0.409, 1.433)	0.23
Males	80	−0.193 (−0.439, 0.053)	−0.22	30	0.325 (−0.219, 0.868)	0.25
Mean arterial pressure						
Combined	157	−**0.196 **(−**0.355,** −**0.037)**	−**0.25**	66	−0.149 (−0.597, 0.299)	−0.11
Females	77	−0.189 (−0.458, 0.081)	−0.19	36	−0.058 (−1.047, 0.931)	−0.03
Males	80	−0.181 (−0.394, 0.033)	−0.22	30	−0.172 (−0.636, 0.293)	−0.16
Carotid reflection index (RIx)						
Combined	155	0.010 (−0.034, 0.054)	0.05	65	0.051 (−0.031, 0.133)	0.20
Females	77	0.005 (−0.075, 0.085)	0.02	35	0.047 (−0.132, 0.225)	0.11
Males	78	0.010 (−0.043, 0.063)	0.06	30	0.051 (−0.044, 0.145)	0.22

cf, carotid-femoral; CVD, cardiovascular disease; PWV, pulse wave velocity **Bold** denotes V̇o_2peak_ is significant contributor to vascular outcome at *P* < 0.05. All analyses in sample without CVD risk factors include covariate adjustment for age, sex (except when analyzed separately), body mass index (BMI), and modality. All analyses in sample with CVD risk factors include covariate adjustment for age, sex (except when analyzed separately), and BMI.

#### Adults with CVD risk factors.

V̇o_2peak_ was inversely associated with cf PWV in the combined sample with CVD risk factors (*P* = 0.01), driven by a significant inverse association among males only (*P* = 0.04) and trend among females (*P* = 0.09; Table 4). V̇o_2peak_ was not significantly associated with MCA conductance, pulsatile damping, carotid stiffness, forward wave energy (*W*1), carotid pulse pressure, mean arterial pressure, or carotid wave reflection (RIx) in males or females (separate or combined).

## DISCUSSION

The major findings of this study include: *1*) V̇o_2peak_ was inversely related to MCA pulsatility in females but not in males without CVD risk factors, and unrelated to MCA mean velocity in males/females combined or separately in the fully adjusted models, *2*) among adults with CVD risk factors, V̇o_2peak_ was positively associated with MCA pulsatility in females, but not associated among males, and *3*) after statistical adjustments for covariates among adults without CVD risk factors, higher V̇o_2peak_ was associated with greater MCA conductance and pulsatile damping, and lower large artery stiffness, forward wave energy, and mean arterial pressure in the combined sample of males and females, with sex-specific associations in large artery stiffness and carotid pulse pressure. For adults with CVD risk factors, V̇o_2peak_ was only inversely associated with aortic stiffness (driven by significant relations among males specifically). Taken together, these data indicate cardiorespiratory fitness appears to target different contributors to cerebrovascular hemodynamics in males versus females without CVD risk factors. These novel findings suggest that the sensitivity of the cerebrovasculature to the beneficial effects of cardiorespiratory fitness may be altered by sex and the presence of modifiable CVD risk factors such as obesity, or medicated hypertension/dyslipidemia.

### Cardiorespiratory Fitness, Cerebral Hemodynamics, and Novel Vascular Contributors to Cerebral Hemodynamics among Adults With Modifiable CVD Risk Factors

We noted no significant association between V̇o_2peak_ and MCA mean velocity, and an inverse relationship between V̇o_2peak_ and MCA pulsatility among females without CVD risk factors but not males in our fully adjusted models. Despite slight differences in unadjusted/age-adjusted models, our fully adjusted models are quite similar to Zeller et al. (11). As such, we largely reproduced the findings from Zeller et al.’s (11) fully adjusted models using a separate, independent sample with a more evenly distributed (particularly among middle-age, see Fig. 1), albeit slightly smaller age range, and fewer individuals with high MCA pulsatility that could have pulled the strength of the relationship between fitness and MCA pulsatility. Indeed, Zeller et al. (11) had ∼18 individuals with MCA pulsatility values >1.0 [highest value ∼1.55, greater than levels seen in hypertensive adults with cognitive impairment (15)], compared with our nine individuals >1.0 (highest value = 1.13). These elevated MCA PI values within Zeller’s sample could have increased the slope between V̇o_2peak_ and MCA PI and contributed to their higher *R*^2^ values for males (0.18) and females (0.40) compared with the smaller *R*^2^ values observed herein. Taken together, these data indicate similar sex-specific relations of cardiorespiratory fitness and MCA pulsatility among females without CVD risk factors, but not males, exhibiting a weak but beneficial relation between greater fitness and lower MCA pulsatility (11).

We further examined sex-specific relations between cardiorespiratory fitness and novel vascular contributors to cerebral mean velocity and pulsatile hemodynamics to expand upon Zeller et al.’s recent work (11). We found V̇o_2peak_ was positively associated with MCA conductance and mean arterial pressure in adults without CVD risk factors, but not in sex-specific analyses, suggestive of modest effects not detectable in our smaller subgroup analyses. Greater cardiorespiratory fitness was also associated with greater cerebral pulsatile damping and lower forward wave energy in males and females without CVD risk factors. Theoretically, greater cerebral (MCA) conductance over time would benefit greater cerebral blood flow, whereas lower forward wave energy and greater pulsatile damping would support beneficial reductions in cerebral pulsatility (12). Ultimately, these beneficial associations between cardiorespiratory fitness and forward wave energy and pulsatile damping were consistent between sexes and may not explain sex-specific relations between greater cardiorespiratory fitness and reduced cerebral pulsatility observed solely among females without CVD risk factors.

Among adults without CVD risk factors, we noted sex-specific, inverse associations between V̇o_2peak_ and *1*) aortic stiffness among males only, and *2*) carotid stiffness and pulse pressure among females only. Inverse associations between cardiorespiratory fitness and aortic stiffness among males, but not females, have been shown cross sectionally previously (31) and may reflect a combination of sex differences in vascular adaptations to regular aerobic exercise (32), or differential sensitivity to obesity/weight change. Aortic stiffness is more sensitive to changes in weight among females than males (33) that may not change as readily among exercising females (34, 35), thereby rendering aortic stiffness more sensitive to cardiorespiratory fitness/regular exercise among males. Carotid stiffness, however, was inversely associated with cardiorespiratory fitness in females only and may reflect the beneficial association of fitness with lower carotid pulse pressure within this group. Lower carotid pulse pressure would be expected to reduce stiffness (36) [and vice versa owing to inherently bidirectional relationship (37)] and could attenuate forward energy wave propagation into the cerebrovasculature (38). Ultimately, carotid pulse pressure is a strong contributor to cerebral pulsatility (12, 39) and brain health (40) regardless of its mechanistic origin, and may be a key mechanism underlying the inverse association between V̇o_2peak_ and MCA PI observed only in females without CVD risk factors.

### Cardiorespiratory Fitness, Cerebral Hemodynamics, and Novel Vascular Contributors to Cerebral Hemodynamics among Adults With Modifiable CVD Risk Factors

We additionally explored the association between cardiorespiratory fitness, and cerebrovascular hemodynamics and their vascular contributors in a subset of participants (*n* = 66) with modifiable CVD risk factors (medicated-hypertension/-dyslipidemia, or BMI ≥ 30 kg/m^2^). Our data indicate that associations between cardiorespiratory fitness and cerebrovascular hemodynamics are attenuated and potentially reversed among individuals with CVD risk factors. Greater fitness among females with CVD risk factors was associated with greater MCA pulsatility after adjusting for age and BMI. This paradoxical relation requires additional exploration but may suggest a mismatch between vascular adaptations to regular exercise and detrimental effects of risk factor burden, or reflect apparently lower cerebral pulsatile damping among females with CVD risk factors. Greater fitness among adults with CVD risk factors was only associated with lower aortic stiffness and was driven by a significant association among males, specifically (although a weaker relation appeared present among females). Although the beneficial relation between fitness and aortic stiffness would benefit brain health over time among males with CVD risk factors, these data broadly indicate that protective associations between fitness and cerebrovascular hemodynamics are less evident among adults with the presence of select modifiable CVD risk factors. As such, having high cardiorespiratory fitness may not fully “rescue/protect” cerebrovascular hemodynamics among individuals with modifiable CVD risk factors (41).

The lack of association between fitness and cerebrovascular hemodynamics among adults with modifiable CVD risk factors could be due to smaller sample size (*n* = 66, 36 females) and/or clustering of V̇o_2peak_ values on the lower spectrum within our sample (Fig. 1*B*). Unsurprisingly, there were a limited number of individuals with CVD risk factors and a V̇o_2peak_ > 40 mL/kg/min. This smaller distribution of V̇o_2peak_ values may stem from the truncated age distribution within our sample with modifiable CVD risk factors (majority of sample >40 yr of age). The absence of younger individuals with CVD risk factors in our sample may reduce the ability to detect the generally weak association between V̇o_2peak_ and cerebral hemodynamics within our CVD risk factor group by limiting the distribution of either *1*) V̇o_2peak_ values (younger individuals tend to have higher V̇o_2peak_) or *2*) vascular outcomes [younger individuals tend to have less stiff arteries, greater pulsatile damping/cerebral blood velocity etc. (12)].

There is evidence, however, that modifiable risk factors and their common pharmaceutical treatments may alter relations between cardiorespiratory fitness and cerebrovascular hemodynamics by influencing vascular adaptations to regular exercise. Indeed, antihypertensive/lipid-lowering medication could attenuate exercise/vascular adaptations by attenuating progression of exercise load (intensity/duration) (20). Large artery stiffness in general may be resistive to change with aerobic exercise training in adults with hypertension (42) and obesity (43), with little influence of antihypertensive (42), but potentially limiting effects of lipid-lowering, medication (44). Cerebral hemodynamics may improve with aerobic exercise training among samples that include a portion of the sample with modifiable risk factors (not necessarily exclusively, however) (45–47). One substantial issue is that cross-sectional and exercise-training studies in this area often exclude individuals using antihypertensive/lipid-lowering medication (47–50) to better isolate the unconfounded physiological effects of cardiorespiratory fitness/exercise training. Although this strengthens internal validity, it weakens external validity when trying to align findings with at-risk, medicated populations that should benefit the most from the protective effects of regular exercise and cardiorespiratory fitness.

The apparent attenuated relationship between cardiorespiratory fitness and cerebral hemodynamics within the sample of adults with CVD risk factors may have also been influenced by the effects of menopause (∼55% of females within this group were postmenopausal). Menopause is associated with increased CVD (51) and cognitive disease risk (52, 53) owing to the loss of the cardio/cerebro-protective effects of estrogen that accelerate vascular (54) and brain aging. Although the influence of menopause on resting cerebral hemodynamics is somewhat unclear (55), the protective effects of cardiorespiratory fitness on cerebrovascular hemodynamics may be attenuated within postmenopausal females, specifically. There is evidence that the benefits of aerobic exercise training are dependent on estrogen, with postmenopausal females experiencing attenuated vascular benefits of aerobic exercise training/cardiorespiratory fitness (32, 56, 57), although this is not universal (58, 59). Importantly, hormone replacement therapy may help reduce cerebral pulsatility (60) and restore beneficial vascular adaptations to aerobic exercise training in postmenopausal females (61, 62). As such, the attenuated association between cardiorespiratory fitness and cerebrovascular hemodynamics among the females with CVD risk factors may be influenced by the prevalence of menopause within this group and its concomitant effect on sex hormones and the vascular benefits of exercise. Ultimately, our data highlight the need to better understand the influence of modifiable CVD risk factors, their respective pharmaceutical treatments, and the influence of menopause on the protective relationship between cardiorespiratory fitness and the cerebrovasculature.

### Limitations and Considerations

Our operational definition of CVD risk factors included key underlying CVD risk factors (obesity, medicated hypertension, or medicated dyslipidemia), but was nonetheless selective and did not consider all CVD risk factors broadly or equitably. We did not comprehensively assess/confirm all CVD risk factors (e.g., family history), relied on self-report and medication use rather than direct measurement of blood lipids, and excluded potent risk factors such as prior CVD events, smoking, and type II diabetes. Thus, our findings may not apply to all CVD risk factors but are limited to those with obesity, medicated-hypertension/dyslipidemia that are still of critical importance since these risk factors impact nearly half of adults (63) and contribute to the development of additional risk factors (e.g., type II diabetes). More detailed characterization of the influence of all CVD risk factors (including type II diabetes, diagnosed CVD/prior events, and nonpharmaceutically treated hypertension/dyslipidemia) on relations between fitness and cerebral hemodynamics in a larger sample is warranted. In addition, we did not have a sample large enough to dissect our CVD risk factor group based on pharmaceutical treatment types or number of risk factors to further examine their effects. It is feasible that obesity and antihypertensive/lipid-lowering medication use may alter the effects of cardiorespiratory fitness differently and thus further work is necessary to untangle their independent and combined effects on the relationship between fitness and cerebrovascular hemodynamics in both sexes. Although our sample includes females from across the menopausal transition, more detailed work must be done to characterize the relations between menopausal status (specifically perimenopause), sex hormones and hormone replacement therapy, cardiorespiratory fitness, and cerebrovascular hemodynamics since this transition period appears critical for substantial changes in vascular hemodynamics and cardiovascular health. Future work should additionally examine resistance exercise, which remains underexplored despite recent data that highlights its potential to improve cerebrovascular hemodynamics (64, 65).

The variation in MCA PI explained by V̇o_2peak_ was generally small (*R*^2^ <0.10), thus cardiorespiratory fitness appears to have a weak direct relationship with MCA PI. The benefits of cardiorespiratory fitness on the brain may accumulate through a combination of weak-to-modest benefits on different aspects of/contributors to cerebrovascular hemodynamics (e.g., improved large artery stiffness, pulsatile damping, cerebrovascular conductance, energy wave transmission, and carotid pulse pressure). In addition, although our comprehensive assessment of mechanistic cerebrovascular hemodynamics is a strength of this study and variables were selected based on our prior work (12) and examined in a priori, sex-specific subgroups based on the study by Zeller et al. (11), interrogation of multiple vascular mechanisms in multiple groups could increase type I error risk. As such, some caution is warranted in interpreting our results owing to the apparent small statistical effects of cardiorespiratory fitness on cerebral hemodynamics, and examination of sex-specific relations within multiple cerebrovascular parameters. Future work examining cardiorespiratory fitness and cerebral hemodynamics should interrogate *1*) additional cerebral vessels or create composite, global cerebral pulsatility indices, *2*) additional levels of pulsatile damping (e.g., internal carotid, vertebral arteries), and *3*) carotid pulse pressure and its contributors as a key, mechanistic gatekeeper of changes in cerebral pulsatility.

### Conclusions

Greater V̇o_2peak_ was associated with small but beneficial changes in cerebrovascular hemodynamics after covariate adjustments in adults without CVD risk factors (greater conductance, pulsatile damping, and lower forward wave energy, mean arterial pressure), and among females was specifically associated with lower cerebral pulsatility, carotid stiffness, and carotid pulse pressure. We noted attenuated relations between V̇o_2peak_ and cerebrovascular hemodynamics among adults with CVD risk factors, with associations between greater V̇o_2peak_ and lower aortic stiffness, among males, and greater MCA pulsatility in females. Our data *1*) indicate that maintaining cardiorespiratory fitness may be one aspect of an integrated lifestyle approach to maintaining optimal cerebral blood flow among adults without CVD risk factors, particularly females, and *2*) underscore the importance of further examining whether modifiable CVD risk factors (and their common pharmaceutical treatments) attenuate or alter the protective associations between cardiorespiratory fitness and cerebrovascular hemodynamics.

## SUPPLEMENTAL DATA

10.6084/m9.figshare.20489706 Supplemental Table S1: https://doi.org/10.6084/m9.figshare.20489706.

## GRANTS

The data presented in this manuscript were funded in part by the American College of Sports Medicine Foundation Research Grant (to W. K. Lefferts); American Heart Association Predoctoral Fellowships 16PRE31220031 (to W. K. Lefferts) and 19PRE34380420 (to E. C. Lefferts); Syracuse University (Sydney Young Research Award) (to J. A. Augustine); School of Education Creative Grant Award (to J. A. Augustine); National Heart, Lung, and Blood Institute of the National Institutes of Health Grant T32HL134634 (to W. K. Lefferts); and Iowa State University College of Human Sciences (Faculty Seed Grant) (to W. K. Lefferts).

## DISCLAIMERS

The content is solely the responsibility of the authors and does not necessarily represent the official views of the National Institutes of Health.

## DISCLOSURES

No conflicts of interest, financial or otherwise, are declared by the authors.

## AUTHOR CONTRIBUTIONS

W.K.L. conceived and designed research; W.K.L., J.A.A., K.S.H., and E.C.L. performed experiments; W.K.L., C.M.W., S.E.M., and E.C.L. analyzed data; W.K.L., C.M.W., S.E.M., and E.C.L. interpreted results of experiments; W.K.L. and S.E.M. prepared figures; W.K.L., C.M.W., and S.E.M. drafted manuscript; W.K.L., C.M.W., S.E.M., J.A.A., K.S.H., and E.C.L. edited and revised manuscript; W.K.L., C.M.W., S.E.M., J.A.A., K.S.H., and E.C.L. approved final version of manuscript.
